# Kappa-Opioid Receptors in the Caudal Nucleus Tractus Solitarius Mediate 100 Hz Electroacupuncture-Induced Sleep Activities in Rats

**DOI:** 10.1155/2012/715024

**Published:** 2012-02-08

**Authors:** Chiung-Hsiang Cheng, Pei-Lu Yi, Han-Han Chang, Yi-Fong Tsai, Fang-Chia Chang

**Affiliations:** ^1^Department of Veterinary Medicine, School of Veterinary Medicine, National Taiwan University, No. 1, Sec. 4, Roosevelt Road, Taipei 10617, Taiwan; ^2^Department of Sports, Health & Leisure, College of Sports Knowledge, Aletheia University, Matou Campus, No. 70-11, Beishiliao, Madou Dist., Tainan City 72147, Taiwan; ^3^Graduate Institute of Brain and Mind Sciences, College of Medicine, National Taiwan University, No. 1, Sec. 1, Ren-Ai Road, Taipei 10051, Taiwan; ^4^Graduate Institute of Acupuncture Science, College of Chinese Medicine, China Medical University, No. 91 Hsueh-Shih Road, Taichung 40402, Taiwan

## Abstract

Previous results demonstrated that 10 Hz electroacupuncture (EA) of Anmian acupoints in rats during the dark period enhances slow wave sleep (SWS), which involves the induction of cholinergic activity in the caudal nucleus tractus solitarius (NTS) and subsequent activation of opioidergic neurons and *μ*-receptors. Studies have shown that different kinds of endogenous opiate peptides and receptors may mediate the consequences of EA with different frequencies. Herein, we further elucidated that high-frequency (100 Hz)-EA of Anmian enhanced SWS during the dark period but exhibited no direct effect on rapid eye movement (REM) sleep. High-frequency EA-induced SWS enhancement was dose-dependently blocked by microinjection of naloxone or *κ*-receptor antagonist (*nor*-binaltorphimine) into the caudal NTS, but was affected neither by *μ*- (naloxonazine) nor *δ*-receptor antagonists (natatrindole), suggesting the role of NTS *κ*-receptors in the high-frequency EA-induced SWS enhancement. Current and previous results depict the opioid mechanisms of EA-induced sleep.

## 1. Introduction

Neuropeptides, along with neurotransmitters, mediate various underlying mechanisms of neural functions and behaviors (e.g., opioid peptides in pain control [1], corticotrophin-releasing hormone (CRH) in stress-related behavior and sleep-wake regulation [2], hypocretin in feeding behavior and in the maintenance of vigilance states [3], etc.). Discovery of endogenous opioid peptides, including *β*-endorphin, dynorphin, enkephalin, and endomorphin, in the central nervous system (CNS) reveals the mysterious actions of acupuncture, especially in its analgesic effect. It had first been demonstrated that the acupuncture-induced analgesic effect could be blocked by a broad-spectrum opioid receptor antagonist naloxone in both humans and mice [4, 5], implicating the role of endogenous opioid peptides. Cheng and Pomeranz had revealed that relatively low doses of naloxone only block the analgesic effect induced by low frequency (4 Hz) of electroacupuncture (EA) stimulation, but not the consequence induced by high frequency (200 Hz) of EA [6], suggesting that the low frequency, rather than the high frequency, of EA increases the release of endogenous opioids. Nevertheless, Han and his colleagues have further shown that the increase of endogenous opioids mediates the analgesic effects induced by both the low-frequency and high-frequency EA stimuli by employing distinct opioid receptor subtype-specific antagonists [7, 8]. While *μ*- and *δ*-opioid receptors in the spinal cord are dominant in the low-frequency EA-induced analgesia, *κ*-opioid receptors contribute to the high-frequency EA effects [7, 8]. Radioimmunoassay of spinal perfusates from rats receiving various frequencies of EA stimulations further indicates that 2 Hz EA enhances enkephalin (a mixed *μ*- and *δ*-opioid receptor agonist) immunoreactivity (IR), but not the dynorphin (*κ*-opioid receptor agonist) IR. In contrast, 100 Hz EA increases dynorphin IR rather than enkephalin IR [9].

Our previous results have shown that 10 Hz EA at Anmian (EX17) acupoints increases slow wave sleep (SWS) in rats, which involves the induction of cholinergic activity in the caudal nucleus tractus solitaries (NTS) [10, 11]. The NTS is located in the dorsomedial medulla oblongata. Ascending projections from the NTS are traced through the lateral and dorsal tegmentum and periventricular gray up to the rostral pons and midbrain and terminate in the parabrachial nucleus, which in turn projects to the thalamus, hypothalamus, preoptic area, bed nucleus of the stria terminalis, amygdala, and the frontal cortex, regions commonly belonging to the visceral-limbic forebrain [12, 13]. From these anatomical data, it does not appear that the predominant effect of the NTS is via the reticular-activating system but instead is via limbic forebrain structures, which are implicated in the sleep regulation. Furthermore, the low-frequency electrical stimulation of the medullary reticular formation, particularly the dorsal reticular formation and the caudal NTS, produces cortical synchronization indicative of SWS in an awake animal [14]. Conversely, lesions of the dorsal reticular formation and of the NTS produced desynchronization of the EEG in a sleeping animal [15]. These results all suggest that the existence of neurons in the NTS is involved in generating sleep. Furthermore, microinjection of morphine into the NTS provokes an enhancement of SWS and this effect is blocked by naloxone [16], suggesting the somnogenic effect of opioidergic system in the NTS. The involvement of opioidergic system in EA's therapeutic indications other than the analgesia (e.g., insomnia) has been less discussed in literature. Our previous study has revealed that 10 Hz (low frequency) EA stimulation of Anmian acupoints increases the concentrations of *β*-endorphin in the brainstem, which consequently enhances SWS through activation of the *μ*-opioid receptors, rather than the *δ*- and *κ*-opioid receptors, in the caudal NTS [11]. However, it has never been determined whether different frequencies of EA stimulations at Anmian acupoints activate distinct opioid receptors in the NTS. This current study was designed to clarify what type(s) of opioid receptor is (are) involved in high-frequency (100 Hz) EA-induced sleep alterations.

## 2. Materials and Methods

### 2.1. Pharmacological Agents

Stock solutions of a broad spectrum opioid antagonist (naloxone hydrochloride (Tocris, Bristol, UK)), a *μ*-receptor antagonist (naloxonazine dihydrochloride (Tocris)), a *δ*-receptor antagonist (naltrindole hydrochloride (Tocris)), and a *κ*-receptor antagonist (*nor*-binaltorphimine dihydrochloride (Tocris)) were dissolved in pyrogen-free saline (PFS). The stock solutions were stored at 4°C until use. Our previous results and others have indicated that the appropriate microinjection dosage for naloxonazine, naltrindole, and *nor*-binaltorphimine to selectively block *μ*-, *δ*-, and *κ*-opioid receptors, without interaction with other opioid receptor subtypes, is within 20 *μ*g [11, 17, 18]. In current study, naloxone, naloxonazine, naltrindole, and *nor*-binaltorphimine were microinjected at three different doses, 0.1, 1.0, and 10 *μ*g/*μ*L. The total volume used for each microinjection was 1 *μ*L.

### 2.2. Animals

Male Sprague-Dawley rats (250–300 g; National Laboratory Animal Breeding and Research Center, Taiwan) were used in this study. Rats were anesthetized by intraperitoneal injection with ketamine/xylazine (87/13 mg/kg) and were given an analgesic (1 mg/rat morphine) and an antibiotic (5000 IU/rat penicillin G benzathine) to reduce pain and avoid infection. Rats were surgically implanted with three electroencephalogram (EEG) screw electrodes as previously described [19] and a microinjection guide cannulae directed into the caudal NTS (AP, -13.30 mm from bregma; ML, 1.2 mm; DV, 8.2 mm relative to bregma). The coordinates were adopted from the Paxinos and Watson rat atlas [20]. Two unilateral screw EEG electrodes were placed over the right hemisphere of the frontal and parietal cortices, and a third EEG electrode was placed over the cerebellum and served to ground the animal to reduce signal artifacts. Insulated leads from EEG electrodes were routed to a Teflon pedestal (Plastics One, Roanoke, VA, USA). The Teflon pedestal was then cemented to the skull with dental acrylic (Tempron, GC Co., Tokyo, Japan). The incision was treated topically with polysporin (polymixin B sulfate-bacitracin zinc), and the animals were allowed to recover for seven days prior to the initiation of experiments. The rats were housed separately in individual recording cages in the isolated room, in which the temperature was maintained at 23 ± 1°C and the light : dark rhythm was controlled in a 12 : 12 h cycle (40 Watt × 4 tubes illumination). Food (5001 rodent diet, LabDiet) and water were available *ad libitum*. All procedures performed in this study were approved by the National Taiwan University Animal Care and Use Committee.

### 2.3. Experimental Protocol

On the 2nd postsurgical day, the rats were connected to the recording apparatus (see below) via a flexible tether. As such, the rats were allowed relatively unrestricted movement within their own cages. Three groups of rats were used in the study as follows: *group 1 *(*n* = 8) was used to determine the effects of opioid receptor antagonist (naloxone) on 100 Hz EA-induced alterations in sleep; *group 2 *(*n* = 8) was used to depict the effects of *μ*-receptor antagonist (naloxonazine) on 100 Hz EA-induced sleep alterations; *group 3 *(*n* = 8) was used to elucidate the effects of *δ*-receptor antagonist (naltrindole) and *κ*-receptor antagonist (*nor*-binaltorphimine) on 100 Hz EA-induced alterations in sleep. One week after rats had adapted to the 12 : 12-hour light : dark cycle after surgery, 24-hour undisturbed baseline recordings were obtained beginning at dark onset on the 1st recording day in rats from all groups. When 100 Hz EA was given (see later), all rats were lightly anesthetized with one-third of the dose of ketamine/xylazine used in the surgery, after which rat woke up in 20 to 25 minutes. A twenty-minute period of EA stimulation was administered before the onset of the dark period. The anesthetization was given 25 minutes prior to the dark period onset and lasted for 20 minutes. The rationale for carrying out the experiment in the darkness is that rats are active with a lowest level of sleep during the dark period, and a manipulation, if it possesses ability to increase sleep, would significantly augment sleep during the dark period. In contrast, it may not be easy to enhance sleep during the light period when sleep activity is at its highest circadian level. Since we expected to find a sleep enhancement after the 100 Hz EA stimuli at the Anmian (EX17), we therefore manipulated the EA stimulation before the onset of the dark period and analyzed the sleep alteration during the subsequent dark period. The rats in *group 1* were intraperitoneally (IP) administered PFS and microinjected with PFS into the caudal NTS (_ip_PFS + PFS) at 25 minutes prior to the dark onset on two consecutive days, and recordings were obtained for 24 h beginning after the second injection. The effects of anesthesia with the NTS microinjection of PFS (_ip_ketamine + PFS) on sleep were determined after IP injection of ketamine/xylazine and the NTS PFS microinjection on two consecutive days. A 100 Hz sham EA (_ip_ketamine + PFS + sham  EA) was delivered to control for the nonspecific effect of the electrical stimulation, although our previous study had confirmed that no nonspecific effect was observed after the sham EA [10, 11]. The 100 Hz EA stimuli under anesthesia (_ip_ketamine + PFS + EA) were also performed before the dark onset on two consecutive days, and sleep-wake behavior after the second 100 Hz EA stimulation was then determined. Subsequently, three different doses (0.1, 1.0, and 10 *μ*g) of naloxone (_ip_ketamine + naloxone + EA) were administered 25 minutes prior to the dark onset on the second day of 100 Hz EA stimulation, and the 24 h sleep pattern was determined. At least one day without injections was scheduled between each manipulation. The 100 Hz EA stimulus was delivered via the bilateral insertion of stainless needles (32 gauge × 1′′, Shanghai Yanglong Medical Articles Co.) on Anmian (EX17) points in the depth of 2 mm. The stimulus consisted of a train of biphasic pulses (150 *μ*s duration each) of 100 Hz with intensity of 3 mA and was delivered by Functions Electrical Stimulator (Trio 300, I.T.O., Japan). The acupoint “Anmian (EX17)” is located at midpoint between Yifeng (TH 17) and Fengchi (GB 20); Yifeng (TH 17) locates posterior to the lobule of the ear in the depression between the mandible and mastoid process; Fengchi (GB 20) locates in the depression between the upper portion of m. sternocleidomastoideus and m. trapezius in human. The location of Anmian (EX17) in rats is at the relative anatomical location between the sternocleidomastoideus muscle and the splenius capitis muscle, as in the human acupoint map. Sham EA was performed by stimulation of a nonacupoint located at the ventral conjunction between the forelimb and the trunk as previous described [10]. Rats in *group 2* received a similar protocol as those in *group 1*, except that the substance administered was naloxonazine (_ip_ketamine + naloxonazine + EA). Those rats in *group 3* underwent a similar protocol as those in *groups 1 *and *2*, except that the substances administered were naltrindole (_ip_ketamine + naltrindole + EA) and *nor*-binaltorphimine (_ip_ketamine + *nor*-binaltorphimine + EA). There was a week interval between the administrations of three doses of naltrindole and those of three doses of *nor*-binaltorphimine.

### 2.4. Apparatus and Recording

Signals from the EEG electrodes were fed into an amplifier (Colbourn Instruments, Lehigh Valley, PA; model V75-01). The EEG was amplified (factor of 5,000) and analog bandpass filtered between 0.1 and 40 Hz (frequency response: ±3 dB; filter frequency roll off: 12 dB/octave). Gross body movements were detected by custom-made infrared-based motion detectors (Biobserve GmbH, Germany), and the movement activity was converted to a voltage output which was digitized and integrated into 1-s bins. These conditioned signals (EEGs and gross body movements) were subjected to analog-to-digital conversion with 16-bit precision at a sampling rate of 128 Hz (NI PCI-6033E; National Instruments, Austin, TX). The digitized EEG waveform and integrated values for body movement were stored as binary computer files pending subsequent analyses.

Postacquisition determination of the vigilance state was done by visual scoring of 12-s epochs using custom software (ICELUS, Mark R. Opp) written in LabView for Windows (National Instruments). The animal's behavior was classified as either SWS, REM sleep, or waking based on previously defined criteria [21]. Briefly, SWS is characterized by large-amplitude EEG slow waves, high power density values in the delta frequency band (0.5–4.0 Hz), and lack of gross body movements. During REM sleep, the amplitude of the EEG is reduced, the predominant EEG power density occurs within the theta frequency (6.0–9.0 Hz), and there are phasic body twitches. During waking, the rats are generally active. There are protracted body movements. The amplitude of the EEG is similar to that observed during REM sleep, but power density values in the delta frequency band are generally greater than those in theta frequency band.

### 2.5. Statistical Analyses for Experiment Protocol

All values acquired from sleep-wake recording were presented as the mean ± SEM for the indicated sample sizes. One-way analyses of variance (ANOVA) for the duration of each vigilance state (SWS, REM sleep, WAKE) and for sleep architecture parameters were performed, comparing before and after manipulation within subjects, across a certain of time block. An *α* level of *P* ≤ 0.05 was taken as indicating a statistically significant difference. If statistically significant differences were detected, a *Scheffe post hoc* comparison was made to determine which hourly intervals during experimental conditions deviated from values obtained from the same animals during control conditions.

## 3. Results

### 3.1. The Effect of Naloxone on the 100 Hz EA-Induced Alterations in Sleep

**Figure 1:**
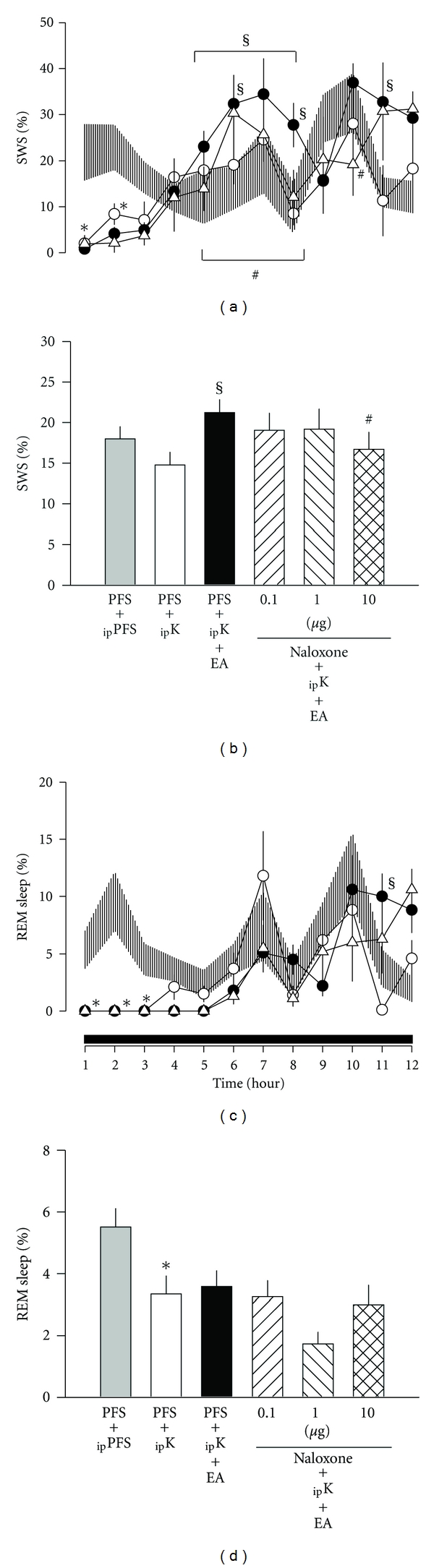
The effects of high-frequency (100 Hz) stimulation by EA at Anmian (EX17) acupoints on vigilance states and the effect of naloxone. Shade area: _ip_PFS + PFS, open circles: _ip_ketamine + PFS, closed circles: _ip_ketamine + PFS + EA, open triangles: _ip_ketamine + naloxone  (10  *μ*g) + EA.*represents a statistically significant difference between the values obtained from _ip_ketamine + PFS and _ip_PFS + PFS. ^§^depicts a statistically significant difference between the values obtained from _ip_ketamine + PFS + EA and _ip_ketamine + PFS. ^#^demonstrates a statistically significant difference between the values obtained from _ip_ketamine + PFS + EA and _ip_ketamine + naloxone + EA. The black horizontal bar on the *x*-axis represents the dark period of the 12 : 12 h light : dark cycle. REM refers to rapid eye movement; SWS slow wave sleep.

Anesthetization of rats for 25 minutes with ketamine/xylazine prior to the dark period suppressed both SWS and REM sleep during the first few hours of the dark period, which is consistent with our previous findings, and the possible mechanisms were discussed later [10, 11]. The percentage of time spent in SWS during the first 2 h period after _ip_ketamine + PFS was decreased from 22.4 ± 3.8% acquired after _ip_PFS + PFS to 5.2 ± 1.4% (*P* < 0.05; Figure 1(a)); however, no significant alteration was detected when SWS during the 12 h of the dark period was analyzed in *group 1* (*F* = 1.269, nonsignificance (n.s.); Figure 1(b)). REM sleep was significantly suppressed from 5.5 ± 0.6% obtained after _ip_PFS + PFS to 3.3 ± 0.6% acquired after _ip_ketamine + PFS during the 12 h of the dark period (*F* = 18.823, *P* < 0.01; Figure 1(d)), especially during the first 3 h after the administrations (Figure 1(c)). Application of 100 Hz sham EA did not alter any aspect of sleep parameters (data not shown), which is similar to our previous observation [10, 11]. Twenty minutes of 100 Hz EA stimuli delivered before the dark period on two consecutive days significantly augmented SWS during the postmanipulation hours 5–8 and hour 11 (Figure 1(a)). Analysis of 12 h dark period revealed that SWS was enhanced from 14.8 ± 1.6% after _ip_ketamine + PFS to 21.2 ± 1.6% after_  ip_ketamine + PFS + EA (*F* = 9.392, *P* < 0.05; Figure 1(b)). The percentage of time spent in SWS during hours 5–8 increased from 17.5 ± 2.1% obtained after _ip_ketamine + PFS to 29.3 ± 2.6% after _ip_ketamine + PFS + EA (*P* < 0.05; Figure 1(a)). The percentage of SWS during the 11h was also enhanced from 28.0 ± 7.3% to 36.9 ± 4.2% (*P* < 0.05; Figure 1(b)). However, REM sleep was not significantly altered by 100 Hz EA (Figures 1(c) and 1(d)). 

**Figure 2:**
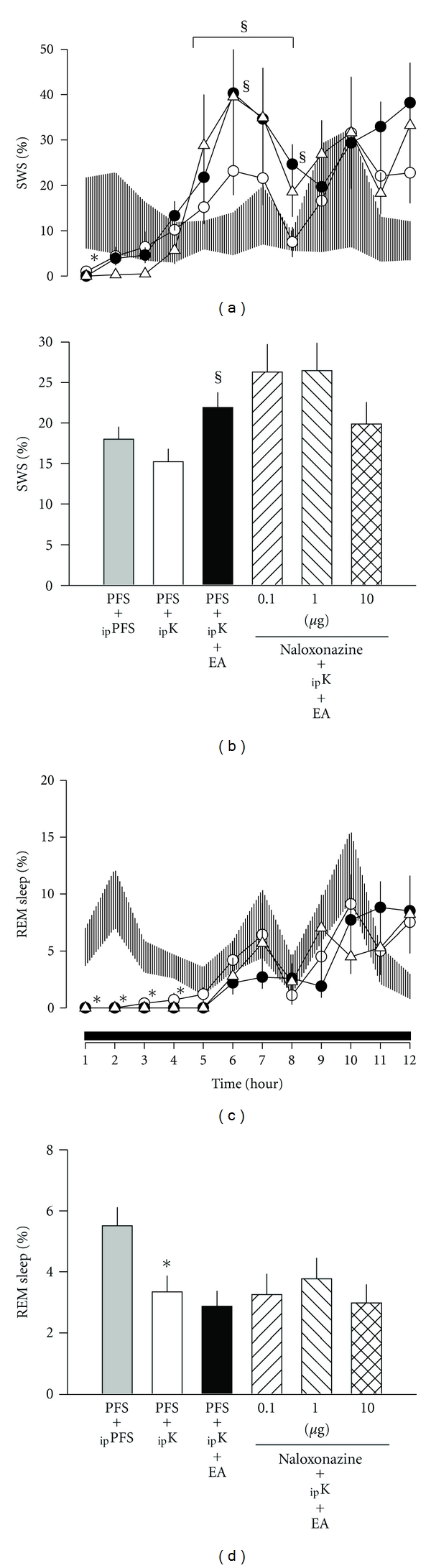
The effects of naloxonazine on 100 Hz EA-induced alterations in sleep. Shade area: _ip_PFS + PFS, open circles: _ip_ketamine + PFS, closed circles: _ip_ketamine + PFS + EA, open triangles: _ip_ketamine + naloxonazine  (10 *μ*g) + EA. *represents a statistically significant difference between the values obtained from _ip_ketamine + PFS and _ip_PFS + PFS. ^§^depicts a statistically significant difference between the values obtained from _ip_ketamine + PFS + EA and _ip_ketamine + PFS.

Administration of three different doses (0.1, 1.0, and 10.0 *μ*g) of naloxone, a broad spectrum opioid antagonist, into the caudal NTS dose-dependently blocked 100 Hz EA-induced increases of SWS, especially during postmanipulation hours 5–8 (Figures 1(a) and 1(b)). Across the entire 12 h dark period recording, SWS was suppressed from 21.2 ± 1.6% after _ip_ketamine + PFS + EA  to 16.7 ± 2.1% after  _ip_ketamine + naloxone  (10.0 *μ*g) + EA (*F* = 18.457, *P* < 0.05; Figures 2(a) and 2(b)), while no significant alteration was detected in REM sleep after administration of nalaxone into the caudal NTS.

### 3.2. The Effect of Naloxonazine on the 100 Hz EA-induced Alterations in Sleep

In *group 2*, the effects of ketamine (suppression of SWS and REM sleep during the first few hours after administrations) and the enhancement of SWS during hours 5–8 after receiving 100 Hz EA stimuli were reproduced as those discovered in *group 1* (Figures 2(a) and 2(c)). Administration of *μ*-opioid receptor antagonist, naloxonazine, into the caudal NTS exhibited no significant effect on the 100 Hz EA-induced SWS enhancement (Figures 2(a) and 2(b)). Naloxonazine also had no effect on REM sleep (Figures 2(c) and 2(d)).

### 3.3. The Effect of Naltrindole on the 100 Hz EA-Induced Alterations in Sleep

**Figure 3:**
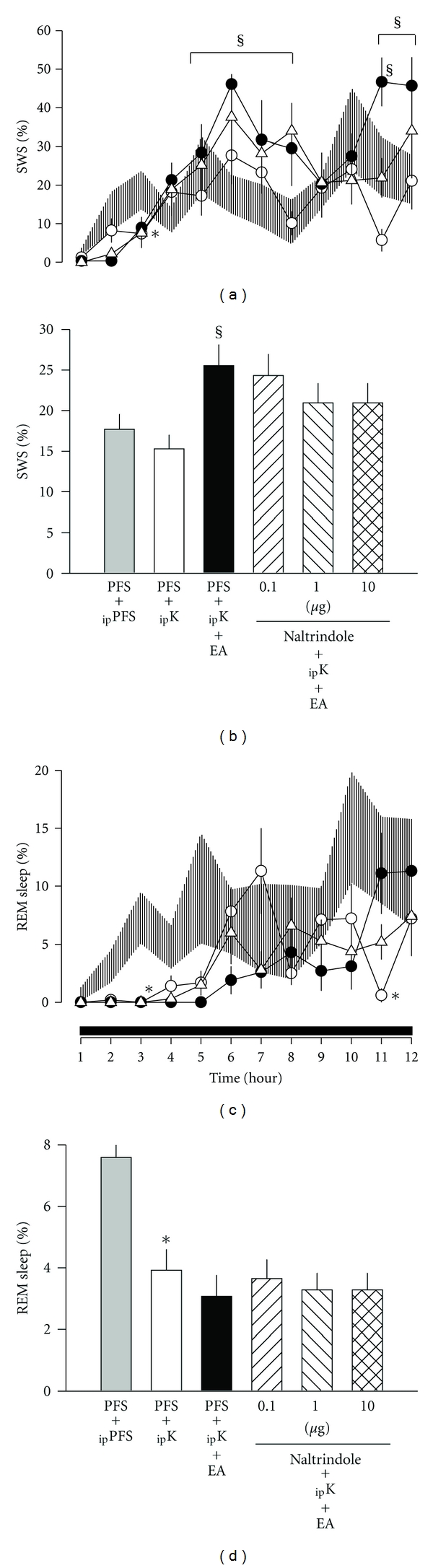
The effects of naltrindole on 100 Hz EA-induced alterations in sleep. Shade area: _ip_PFS + PFS, open circles: _ip_ketamine + PFS, closed circles: _ip_ketamine + PFS + EA, open triangles: _ip_ketamine + naltrindole  (10  *μ*g) + EA. *represents a statistically significant difference between the values obtained from _ip_ketamine + PFS and _ip_PFS + PFS. ^§^depicts a statistically significant difference between the values obtained from _ip_ketamine + PFS + EA  and _ip_ketamine + PFS.

In *group 3*, the effects of ketamine (suppression of SWS and REM sleep during the first few hours after administrations) and the enhancement of SWS during hours 5–8 and hours 11–12 after receiving 100 Hz EA stimuli were also reproduced as those found in *groups 1 *and *2 *(Figures 3(a) and 3(c)). Administration of *δ*-opioid receptor antagonist, naltrindole, into the caudal NTS exhibited no significant effect on the 100 Hz EA-induced SWS enhancement (Figures 3(a) and 3(b)). Naltrindole had no further effect on REM sleep (Figures 3(c) and 3(d)).

### 3.4. The Effect of Nor-Binaltorphimine on the 100 Hz EA-Induced Alterations in Sleep

**Figure 4:**
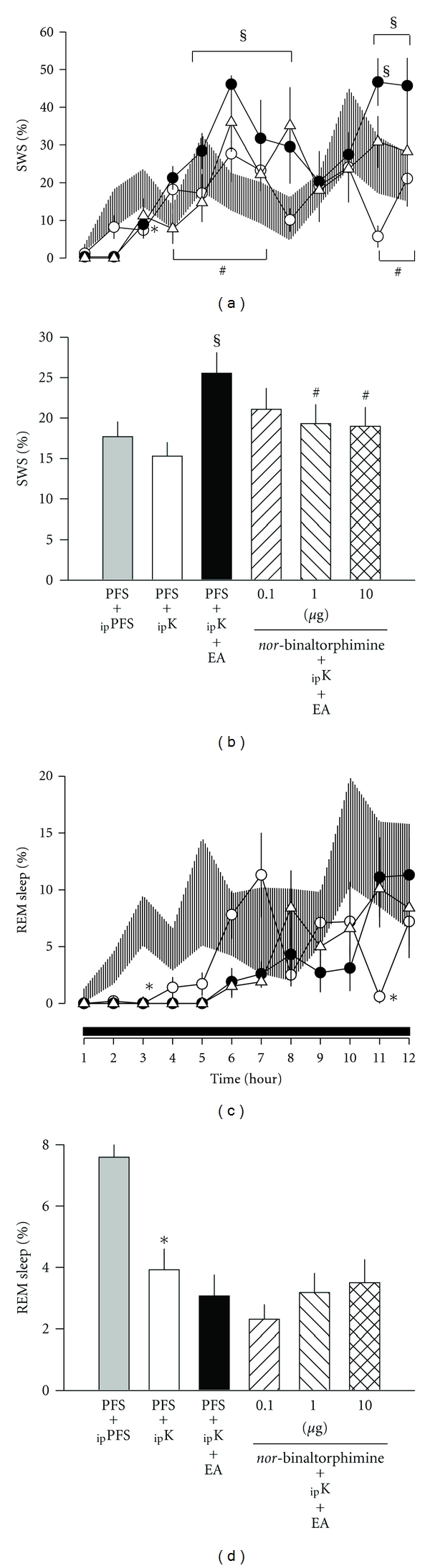
The effects of *nor*-binaltorphimine on 100 Hz EA-induced alterations in sleep. Shade area: _ip_PFS + PFS, open circles: _ip_ketamine + PFS, closed circles: _ip_ketamine + PFS + EA, open triangles: _ip_ketamine + *nor*-binaltorphimine  (10  *μ*g) + EA. *represents a statistically significant difference between the values obtained from _ip_ketamine + PFS and _ip_PFS + PFS. ^§^depicts a statistically significant difference between the values obtained from _ip_ketamine + PFS + EA and _ip_ketamine + PFS. ^#^demonstrates a statistically significant difference between the values obtained from _ip_ketamine + PFS + EA and _ip_ketamine + *nor*-binaltorphimine + EA.

In *group 3*, our results indicate that the enhancement of SWS during hours 5–8 and hours 11-12 induced by 100 Hz EA stimuli was dose-dependently blocked by the *κ*-opioid receptor antagonist, *nor*-binaltorphimine. The percentage of time spent in SWS across the entire 12 h dark period was suppressed from 25.6 ± 2.6% obtained after _ip_ketamine + PFS + EA to 21.1 ± 2.6% (*F* = 3.574, n.s.), 19.3 ± 2.4% (*F* = 9.784, *P* < 0.05), and 19.0 ± 2.4% (*F* = 9.897, *P* < 0.05; Figures 4(a) and 4(b)) after administrations of 0.1, 1.0, and 10 *μ*g of *nor*-binaltorphimine, respectively.

The effect of *nor*-binaltorphimine on blocking the 100 Hz EA-induced SWS enhancement dominantly occurred during hours 4–7 and hours 11-12 (Figure 4(a)). *Nor*-binaltorphimine exhibited no effect on REM sleep (Figures 4(c) and 4(d)).

## 4. Discussion

Acupuncture and electroacupuncture (EA) have been recommended as an alternative medicine for several therapeutic indications by the World Health Organization (WHO), such as alleviation of pain, reduction of inflammation, and management of insomnia. The theory underlying EA is still controversial, although the action of EA has been widely discussed in literature. The discovery of endogenous opioid peptides, including enkephalin, *β*-endorphin, dynorphin, and endomorphin, since 1970s enhances the investigation of underlying mechanisms of EA, especially in the EA-induced analgesia. Three main receptor subtypes of the opioid receptors, including the *μ*-, *δ*-, and *κ*-opioid receptors, in the spinal cord have been indicated involve in the mechanisms of EA-induced analgesia. Endomorphin and dynorphin are, respectively, considered as the relatively pure *μ*- and *κ*-opioid receptor agonists [22, 23], while enkephalin and *β*-endorphin are mixed *μ*- and *δ*-opioid receptor agonists (review [1, 24]). Han and his colleagues have revealed that low-frequency (2 Hz) EA increases met-enkephalin, but not dynorphin, in the spinal cord; while high-frequency (100 HZ) EA increases the release of dynorphin rather than that of met-enkephalin [9]. The stimulation of EA between low and high frequencies (e.g., 15 Hz) activates both enkephalins and dynorphins [9]. They further demonstrated that the analgesic effect induced by low-frequency EA stimulation is mediated by *μ*- and/or *δ*-opioid receptors; in contrast, high-frequency EA-induced analgesia is mediated by *κ*-opioid receptors [7, 8]. These observations suggest that different endogenous opioid peptides would be released and act on distinct opioid receptors in the spinal cord under different stimulating conditions of EA. However, the involvement of opioid receptors in sleep alteration under different stimulating conditions of EA remains undetermined.

We previously demonstrated that 10 Hz EA stimuli of Anmian (EX17) acupoints in anesthetized rats for 20 minutes prior to the beginning of the dark period of the light : dark cycle in two consecutive days enhance SWS during the subsequent dark period [10, 11]. Our results also implicated that 10 Hz EA-induced SWS enhancement may be mediated, in part, by vagal cholinergic afferents to the caudal NTS [10, 11]. There are two anatomically distinct *β*-endorphin pathways in the brain; the major pathway originates in the arcuate nucleus, and the minor one is in the area of the NTS of the caudal medulla [25]. Furthermore, dynorphin is also expressed in neurons of the brainstem NTS [26]. The anatomical distributions of opioid receptors and peptides in the CNS have been well described by Mansour et al. [27]. The neurons containing proopiomelanocortin (POMC), proenkephalin, and prodynorphin are abundant in the NTS [27]. The *μ*- and *κ*-opioid receptors are localized in the caudal NTS with a highly density; however, little or no *δ*-opioid receptor can be observed in the brainstem, including the NTS [27]. Our previous results have depicted that the SWS enhancement after 10 Hz EA of Anmian acupoints is mediated by increasing the concentrations of *β*-endorphin and activation of the *μ*-opioid receptors in the NTS [11]. Herein, we tried to further elucidate whether high-frequency (100 Hz) EA stimulation of Anmian acupoints also elicits similar effect of sleep enhancement and whether the sleep alterations under different EA stimulation conditions are mediated by distinct opioid receptors. Our current results demonstrated that 100 Hz (high-frequency) EA stimuli of Anmian (EX17) acupoints in anesthetized rats for 20 minutes in two consecutive days enhanced SWS, but not REM sleep, during the subsequent dark (active) period. The enhancement of SWS after 100 Hz EA is similar to that after 10 Hz EA stimulation [11], indicating both low-frequency (10 Hz) and high-frequency (100 Hz) EA stimuli possess ability to alter SWS in the same direction. Application of naloxone, a broad spectrum of opioid receptor antagonist, was used to determine the involvement of NTS opioidergic receptors in the 100 Hz EA-induced increase of SWS. We found that administration of naloxone directly into the caudal NTS dose-dependently blocked 100 Hz EA-induced SWS enhancement during the dark period, implicating the involvement of endogenous opiates in the caudal NTS. A potential role for three major opioid receptors, *μ*-, *δ*-, and *κ*-receptors, was then determined by application of specific receptor antagonists. Our results demonstrated that *nor*-binaltorphimine, a *κ*-opioid receptor antagonist, exhibited a similar dose-dependent effect on blocking 100 Hz EA-induced SWS enhancement as that of naloxone; whereas naloxonazine (a *μ*-opioid receptor antagonist) and naltrindole (a *δ*-opioid receptor antagonist) had no effect. These observations combined with our previous results—*μ*-opioid receptors mediate 10 Hz EA-induced SWS enhancement [11]—suggest that distinct opioid receptors in the NTS involve different stimulation frequencies of EA-induced SWS enhancement, which is similar to the underlying mechanisms of EA-induced analgesia in the spinal cord as reported by Han and his colleagues [1, 7]. Strategy by employing pharmacological blockade to elucidate the involvement of particular opioid receptors in 100 Hz EA-induced sleep alteration is appropriate. However, it would be of interest to mimic the EA-induced sleep alteration by microinjection of *κ*-receptor agonist, for example, dynorphin, into the NTS in future.

In order to perform the 100 Hz EA stimulation easily, rats were lightly anesthetized. We found that both SWS and REM sleep during the first few hours of the dark period were decreased after rats recovered from the ketamine anesthetic. The decreases in SWS and REM sleep were also observed in our previous study [10, 11]. Ketamine, a cyclohexanone derivative, is used clinically as a dissociative anesthetic agent both in humans and animals. Ketamine is a noncompetitive N-methyl-D-aspartate (NMDA) receptor antagonist that blocks cation channels [28]. It has been demonstrated that administration of ketamine or MK-801, another NMDA receptor antagonist, at subanesthetic doses produces a robust, dose-dependent increase in the intensity of *δ*-power of the NREM sleep [29, 30]. Furthermore, the effect of MK-801 by increasing the metabolic rate in the hippocampus and other limbic structures stimulates physiological sleep that is similar to the sleep that follows sleep deprivation, indicating the need of homeostatic recovery [31]. Therefore, the suppression of NREM and REM sleep after recovery from the ketamine anesthetization during the beginning of the dark period may be due to a homeostatic compensation to the previous anesthetic state. However, this explanation needs to be further investigated.

## 5. Conclusion

**Figure 5:**
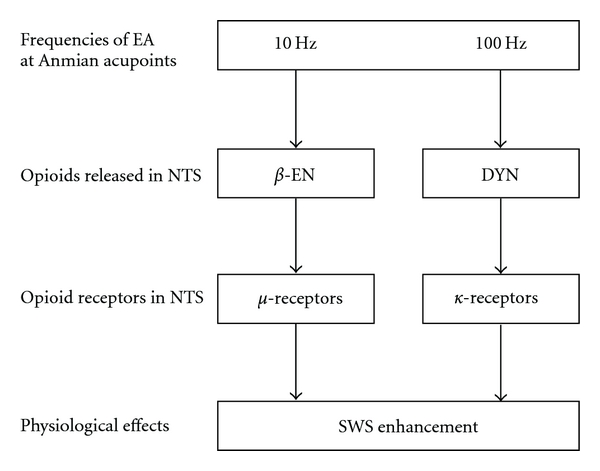
A hypothetical model by which different frequencies of EA Anmian (EX17) alter SWS through different opioid receptors in the caudal NTS. *β*-EN: *β*-endorphin; DYN: dynorphin.

In summary, our current results demonstrated that 100 Hz EA stimuli of Anmian (EX17) acupoints enhance SWS and this enhancement is blocked by naloxone and *nor*-binaltorphimine, implicating the mediation of *κ*-opioid receptors. Compared to our previous observation of involvement of *μ*-opioid receptors in 10 Hz EA-induced SWS enhancement, we concluded that *μ*-opioid receptors in the caudal NTS mediate the low-frequency (10 Hz) EA-induced SWS alteration, while *κ*-opioid receptors mediate the high-frequency (100 Hz) EA-induced SWS enhancement. A diagram elucidating one hypothetical mechanism by which different frequencies of EA at Anmian (EX17) alters sleep is depicted in Figure 5.
